# Supplementary material for: Ongoing Gaps in the Hepatitis C Care Cascade during the Direct-Acting Antiviral Era in a Large Retrospective Cohort in Canada: A Population-Based Study
Source: Viruses. 2024 Mar 1;16(3):389. doi: 10.3390/v16030389 (PMC10975766; doi:10.3390/v16030389)
Supplement: Supplementary file 1 [file viruses-16-00389-s001.zip › viruses-2880543-supplementary.pdf]

Supplementary material

Table S1. List of codes used to identify comorbidities and liver-related disease in hospital, medical visits, and pharmacy dispensations data.

Condition	Codes
**Injection drug use**<br>(2 medical visits or 1 hospitalization or drug-related death)	ICD-9 starting with: 3040, 3047, 3055, 9650, 3042, 3056, 970, 3041, 9694, 3054, 292, 3045, 3046, 3048, 3049, 3053, 3059, 6483, 7960, 9621, 9658, 9663, 9664, 9670, 9684, 9685, 9696, 9698, 9699, 970.<br>ICD-10 exact codes: T400, T401, T402, T403, T404, T406, R781, E8500, T405, R782, T436, T423, T424, T425, T426, T427, T428, V6542, T387, T408, T409, T412, T436, T437, T438, T439, T507; starting with: F11, F14, F13.<br>Drug-related mortality codes (death registry): F11, F12, F13, F14, F15, F16, F19, X40, X41, X42, X43, X44, X60, X61, X62, X63, X64, X85, Y10, Y11, Y12, Y13, Y14, Y40, Y41, Y42, Y43, Y44, Y45, Y46, Y47, Y48, Y49, Y50, Y51, Y52, Y53, Y54, Y55, Y56, Y570, Y571, Y572, Y573, Y574, Y577, Y578, Y579, Y598, Y880, D521, D590, D592, D611, D642, E032, E064, E231, E242, E273, F55, F551, G210, G211, G240, G251, G254, G256, G444, G620, G720, H263, I427, I952, J702, J703, J704, L105, L233, L244,<br>L251, L270, L271, L432, L560, L561, L640, M022, M102, M320, M804, M814, M835, M871, N140,<br>N141, N142, O355, P040, P041, P044, P584, P961, P962, R781, R782, R783, R784, R785, R786, R825,<br>304, 3052, 3053, 3054, 3055, 3056, 3057, 3058, 3059, 292, 9090, 965, 966, 967, 968, 969, 970,<br>971, 972, 973, 974, 975, 976, 977, 978, 979, 6483, 3576.
**Elixhauser comorbidity index (modified)**[a]<br>(presence of at least 1 code)	Starting with :<br>Congestive Heart Failure: 39891, 40201, 40211, 40291, 40401, 40403, 40411, 40413, 40491, 40493, 4254, 4255, 4257, 4258, 4259, 428. Cardiac Arrhythmia: 4260, 42613, 4267, 4269, 42610, 42612, 4270, 4271, 4272, 4273, 4274, 4276, 4278, 4279, 7850, 99601, 99604, V450, V533. Valvular Disease: 0932, 394, 395, 396, 397, 424, 7463, 7464, 7465, 7466, V422, V433. Pulmonary Circulation Disorders: 4150, 4151, 416, 4170, 4178, 4179. Peripheral Vascular Disorders: 0930, 4373, 440, 441, 4431, 4432, 4438, 4439, 4471, 5571, 5579, V434. Hypertension: 401, 402, 403, 404, 405. Paralysis: 3341, 342, 343, 3440, 3441, 3442, 3443, 3444, 3445, 3446, 3449. Other Neurological Disorders: 3319, 3320, 3321, 3334, 3335, 33392, 334, 335, 3362, 340, 341, 345, 3481, 3483, 7803, 7843. Chronic Pulmonary Disease: 4168, 4169, 490, 491, 492, 493, 494, 495, 496, 500, 501, 502, 503, 504, 505, 5064, 5081, 5088. Hypothyroidism: 2409, 243, 244, 2461, 2468. Renal Failure: 40301, 40311, 40391, 40402, 40403, 40412, 40413, 40492, 40493, 585, 586, 5880, V420, V451, V56. Peptic Ulcer Disease excluding bleeding: 5317, 5319, 5327, 5329, 5337, 5339, 5347, 5349. Lymphoma: 200, 201, 202, 2030, 2386. Cancer: 196, 197, 198, 199, 140, 141, 142, 143, 144, 145, 146, 147, 148, 149, 150, 151, 152, 153, 154, 155, 156, 157, 158, 159, 160, 161, 162, 163, 164, 165, 166, 167, 168, 169, 170, 171, 172, 174, 175, 176, 177, 178, 179, 180, 181, 182, 183, 184, 185, 186, 187, 188, 189, 190, 191, 192, 193, 194, 195. Rheumatoid Arthritis/collagen: 446, 7010, 7100, 7101, 7102, 7103, 7104, 7108, 7109, 7112, 714, 7193, 720, 725, 7285, 72889, 72930. Coagulopathy: 286, 2871, 2873, 2874, 2875. Obesity/weight loss/Fluid and Electrolyte Disorders: 2780, 260, 261, 262, 263, 7832, 7994, 2536, 276. Anemia: 2800, 2801, 2808, 2809, 281.
**Diabetes**<br>(2 medical visits within 2 years or 1	ICD-9 starting with: 250.<br>ICD-10 starting with: E10, E11, E12, E13, E14.

hospitalization or 1 prescription for antidiabetic drug)	Pharmacy dispensation common denomination codes: 46300, 47151, 05824, 46862, 47208, 47807, 47832, 47964, 47965, 48017, 48044, 48117, 48134, 48137, 47817, 47715, 48018, 47881, 47867, 47836, 48200, 48085, 04823, 04888, 18296, 18309, 18322, 18335, 18348, 39120, 39133, 39146, 39159, 39172, 39185, 39458, 39484, 39497, 39523, 41655, 43033, 43735, 44151, 44164, 44476, 44489, 44502, 44996, 45405, 45415, 45483, 45511, 45531, 45534, 46322, 46536, 46537, 46538, 46592, 46602, 46603, 46607, 46798, 47004, 47206, 47424, 47426, 47536, 47586, 47615, 47749, 48163, 48174, 48223, 48243, 46568, 47357, 46810, 48013, 48062, 48039, 00091, 01937, 46056, 47329, 46799, 47427, 47652, 04264, 09672, 15184, 46642, 47371, 46678, 47392.
**Major mental health illness diagnosis** (2 medical visits or 1 hospitalization)	ICD-9 starting with: 295, 296, 297, 298, 300, 301, 308, 309, 311.<br>ICD-10 starting with: F20, F21, F22, F23, F24, F25, F28, F29, F30, F31, F32, F33, F34, F38, F39, F40, F41, F42, F43, F44, F45, F48, F60, F61.
**Alcohol use disorder** (2 medical visits or 1 hospitalization)	ICD-9 starting with: 291, 303, 3039, 3050, 3575, 4255, 425, 5353, 7903, 9773, 980, V113<br>ICD-10 starting with: F10, E244, G312, G621, G721, I426, Z502, Z714, K292, T51
**HIV co-infection** (2 medical visits or 1 hospitalization within 2 years OR 2 medical visits or 2 dispensation records of HIV medications within 2 years)	ICD-9 starting with: 042, 043, 044, V08.<br>ICD-10 starting with: B20, B21, B22, B23, B24, R75, Z21.<br>Pharmacy dispensation common denomination codes [any single drug, including Truvada, must be combined with at least one other drug AND treatment must be prescribed for more than 28 days]: 46769, 47422, 47576, 48221, 48026, 48251, 48043, 48295, 48247, 48258, 47724, 48114, 47946, 47895, 48122, 48160, 46520, 47310, 47397, 46714, 46769, 47422, 47576, 48221, 48026, 48251, 48043, 48295, 48247, 48258, 47724, 48114, 47946, 47895, 48122, 48160, 46520, 47310, 47397, 46714, 47605, 47701, 46564, 47324, 46581, 45611, 46514, 47147, 46311, 47171, 47502, 47512, 45589, 45516, 47313, 46461, 46544, 47323, 47727, 46505, 47294, 47876, 47891, 48226, 47479, 47623, 47632, 47543, 46475, 47293, 46313, 47170, 46519, 47158, 47306, 47585, 47167, 46743, 47423, 47984, 47700, 47466, 45611
**Decompensated cirrhosis** (2 medical visits or 1 hospitalization)	ICD-9 starting with: 4560, 4562, 5672, 5713, 5722, 5724, 5728, 7895, 0704.<br>ICD-10 starting with: K652, K704, K721, K729, K767, I850, I982, I9820, I983, R18.
**Hepatocellular carcinoma** (2 medical visits or 1 hospitalization)	ICD-9 : 1550.<br>ICD: C220.

[a]Codes for diabetes, mental health illness, alcohol and drug consumption disorders, HIV, and liver-disease excluded.

Table S2. Validation of the PWID algorithm in Quebec data.

We used a published algorithm for identifying PWID in health administrative data that has been validated in BC and ON (1, 2). To validate this algorithm in Quebec we used a cohort of PWID (2004-2018) from HEPCO (3, 4) for true positives. The true negative reference standard comprised a random population sample from the FIPA database presumed to be non-PWID based on the low prevalence of injection drug use in Quebec (0.3%) (5) matched to the HCV cases on sex, age, and rurality at a 333:1 ratio. This generated a dataset of 829 PWID (HEPCO, 0.3%) and 276,057 individuals from the general population (FIPA, presumed non-PWID, 99.7%). Codes used in medical visits, hospitalization, and mortality data to identify PWID are described in **Table S1.**

Algorithm description	Sensitivity	95% CI	Specificity	95% CI	PPV	NPV	Accuracy
**1 medical visit or 1 hospitalization or drug-related death**	100%	99.54, 100	99.16%	99.12, 99.19	26.24% (24.74, 27.8)	100% (100, 100)	99.16% (99.12, 99.19)

Table S3. Algorithms for treatment initiations not covered by the provincial pharmacy prescription plan.

Quebec pharmacy dispensations data covered 68% of the HCV cohort for the entire study period (1990-2018). Treatment status for the remaining 32% of the cohort was imputed with a multiple logistic regression predictive model developed using the subset of individuals fully covered in a 60%/40% training/validation data split (6). The model was constructed based on the pattern of individuals' RNA tests and genotyping, adjusted for socioeconomic characteristics, medical comorbidities, and liver-related diseases. It showed high discrimination (c-statistic of 0.902), and was 75% sensitive and 88% specific at predicting treatment initiation. The total number of observed and imputed HCV-specific antiviral treatment initiations between 2016-2018 was benchmarked with reported DAA prescriptions during the same period by the Institute for Human Data Science (IQVIA) (7, 8). The date of imputed treatment initiations was imputed based on the dates and patterns of RNA tests. which was 95% accurate to the calendar year of treatment initiation when validated in the observed treatment initiations cohort.

**Treatment initiation algorithm**<br><br><u>Step 1: Multiple logistic regression model</u><br>- Outcome: observed treatment initiations<br>- Main exposure of interest:<br>o Total count of RNA tests on record and a squared term (due to non-linear association)<br>o Total count of genotype tests on record and a squared term (due to non-linear association)<br>o Last RNA test result on record<br>o An interaction term for people who inject drugs and total RNA tests count (to correct for more frequent RNA testing of anti-HCV-negative PWID that could result in higher false-positives among PWID)<br>o Calendar year of HCV diagnosis<br>- Additional baseline characteristics:<br>o Sociodemographic characteristics: sex, age and age squared, immigrant status, PWID status, region of residence, material and social deprivation status<br>o Comorbidities/liver-related disease: decompensated cirrhosis and hepatocellular carcinoma<br><br><u>Step 2: Different cutoffs for predicted probability of treatment initiations by calendar year of HCV diagnosis (data-driven approach to obtain a balance of highest sensitivity and specificity)*</u><br>- 1990-2004: 0.435<br>- 2005-2013: 0.380<br>- 2014: 0.300<br>- 2015: 0.250<br>- 2016-2017: 0.220<br>- 2018: 0.120<br><br>*To account for different follow-up time, changes in RNA monitoring during treatment, and resulting inverse correlation between total count of RNA tests on record and calendar year of HCV diagnosis.
**Date of algorithm-imputed treatment initiation**<br><br>Date of treatment initiation = date of 3rd RNA test on record (data-driven approach to obtain the highest accuracy)<br><br>- If interval between 3rd and 5th RNA test on record greater than 18 months:

- o Date of treatment initiation = date of 5th RNA test on record
- Validation by calendar-year only

Table S4. Characteristics of individuals diagnosed with HCV in 1990-2018 and living in Quebec, Canada as of 2018, in full cohort and imputed cohort.

	**Full Cohort**			**Imputed Cohort**
**Characteristics**	***Total*** (n=42514)	***Linked*** (n=37949)	***Unlinked*** (n=4565)	***Total*** (n=42514)
Total diagnosed	*42514 (100.0)*	*37949 (100.0)*	*4565 (100.0)*	*42514 (100.0)*
Died	*9952 (23.4)*	*9952 (26.2)*		*11075 (26.0)*
Missing			*4565 (100.0)*	
**Living as of 2018**	**27997 (65.9)**	**27997 (73.8)**		**31439 (74.0)**
**Age, mean (SD)**	41.7 (13.1)	41.7 (12.9)	41.6 (14.6)	41.5 (12.9)
**Birth cohort**				
<1945	1870 (5.7)	1594 (5.7)	276 (6.0)	1756 (5.6)
1945-1965	17048 (52.4)	15579 (55.7)	1469 (32.2)	17220 (54.8)
>1965	12087 (37.12)	10824 (38.7)	1263 (27.7)	12463 (39.6)
Missing	1557 (4.8)	-- --	1557 (34.1)	
**Sex**				
Female	10572 (32.5)	9701 (34.7)	871 (19.1)	10700 (34.0)
Male	20540 (63.1)	18296 (65.3)	2244 (49.2)	20739 (66.0)
Missing	1450 (4.5)	-- --	1450 (31.8)	
**Region of residence**				
Urban	21571 (66.2)	18449 (65.9)	3122 (68.4)	20775 (66.1)
Rural	10904 (33.5)	9464 (33.8)	1440 (31.5)	10664 (33.9)
Missing	87 (0.3)	84 (0.3)	3 (0.1)	
**Social deprivation quintile**				
1 (Least deprived)	2828 (8.7)	2828 (10.1)	-- --	3176 (10.1)
2	3470 (10.7)	3470 (12.4)	-- --	3932 (12.5)
3	4296 (13.2)	4296 (15.3)	-- --	4938 (15.7)
4	6508 (20.0)	6508 (23.2)	-- --	7524 (23.9)
5 (Most deprived)	10317 (31.7)	10317 (36.9)	-- --	11869 (37.8)
Missing	5143 (15.8)	578 (2.1)	4565 (100.0)	
**Material deprivation quintile**				
1 (Least deprived)	3892 (12.0)	3892 (13.9)	-- --	4483 (14.3)
2	4191 (12.9)	4191 (15.0)	-- --	4801 (15.3)
3	4932 (15.1)	4932 (17.6)	-- --	5680 (18.1)
4	6051 (18.6)	6051 (21.6)	-- --	6939 (22.1)
5 (Most deprived)	8353 (25.7)	8353 (29.8)	-- --	9536 (30.3)

Missing	5143 (15.8)	578 (2.1)	4565 (100.0)	
**Immigrant status**				
Immigrant	4166 (12.8)	4166 (14.9)	-- --	4862 (15.5)
Non-immigrant	23831 (73.2)	23831 (85.1)	-- --	26577 (84.5)
Missing	4565 (14.0)	-- --	4565 (100.0)	
**Region of origin**				
East Asia And Pacific	714 (17.1)	714 (17.1)	-- --	915 (18.8)
South Asia	408 (9.8)	408 (9.8)	-- --	545 (11.2)
Middle East & North Africa	505 (12.1)	505 (12.1)	-- --	631 (13.0)
Sub Saharan Africa	602 (14.5)	602 (14.5)	-- --	854 (17.6)
High Income Europe	596 (14.3)	596 (14.3)	-- --	783 (16.1)
Middle Income Europe & Central Asia	433 (10.4)	433 (10.4)	-- --	579 (11.9)
Latin America And Caribbean	377 (9.0)	377 (9.0)	-- --	477 (9.8)
United States, Australia And New Zealand	52 (1.2)	52 (1.2)	-- --	78 (1.6)
Missing	479 (11.5)	479 (11.5)	-- --	
**Immigration category**				
Economic	1266 (30.4)	1266 (30.4)	-- --	1682 (34.6)
Family	1209 (29.0)	1209 (29.0)	-- --	1549 (31.9)
Refugee	1156 (27.7)	1156 (27.7)	-- --	1498 (30.8)
Other	57 (1.4)	57 (1.4)	-- --	132 (2.7)
Missing	478 (11.5)	478 (11.5)	-- --	
**Language proficiency**				
French only	1092 (26.2)	1092 (26.2)	-- --	1419 (29.2)
French and English	750 (18.0)	750 (18.0)	-- --	1066 (21.9)
English only	672 (16.1)	672 (16.1)	-- --	884 (18.2)
Neither	1165 (28.0)	1165 (28.0)	-- --	1493 (30.7)
Missing	487 (11.7)	487 (11.7)	-- --	
**Highest education level**				
High school or less	1823 (43.8)	1823 (43.8)	-- --	2445 (50.3)
Certificate/diploma	928 (22.3)	928 (22.3)	-- --	1214 (25.0)
University/Postgraduate	836 (20.1)	836 (20.1)	-- --	1203 (24.8)
Missing	579 (13.9)	579 (13.9)	-- --	
**Years from admission to diagnosis, median (IQR)**	6.6 (1.1-14.5)	6.6 (1.1-14.5)	-- --	6.5 (1.1-14.3)
Missing	478.0 (11.0)	478.0 (11.0)		
**People who inject drugs**				
Yes	12529 (38.5)	12529 (44.7)	-- --	14008 (44.6)
No	15468 (47.5)	15468 (55.3)	-- --	17431 (55.4)
Missing	4565 (14.0)	-- --	4565 (100.0)	
**Comorbidities (at baseline)**				
Major mental health illness				
Yes	7761 (23.8)	7761 (27.7)	-- --	8685 (27.6)
No	20236 (62.1)	20236 (72.3)	-- --	22755 (72.4)
Missing	4565 (14.0)	-- --	4565 (100.0)	
Alcohol use disorder				
Yes	2651 (8.1)	2651 (9.5)	-- --	3095 (9.8)
No	25346 (77.8)	25346 (90.5)	-- --	28344 (90.2)
Missing	4565 (14.0)	-- --	4565 (100.0)	

HBV coinfection				
Yes	867 (2.7)	867 (3.1)	-- --	1002 (3.2)
No	27130 (83.3)	27130 (96.9)	-- --	30437 (96.8)
Missing	4565 (14.0)	-- --	4565 (100.0)	
HIV coinfection				
Yes	981 (3.0)	981 (3.5)	-- --	1272 (4.0)
No	27016 (83.0)	27016 (96.5)	-- --	30167 (96.0)
Missing	4565 (14.0)	-- --	4565 (100.0)	
**Liver-related outcomes**				
Decompensated cirrhosis				
Yes	431 (1.3)	431 (1.5)	-- --	675 (2.1)
No	27566 (84.7)	27566 (98.5)	-- --	30764 (97.9)
Missing	4565 (14.0)	-- --	4565 (100.0)	
Hepatocellular carcinoma				
Yes	29 (0.1)	29 (0.1)	-- --	251 (0.8)
No	27968 (85.9)	27968 (99.9)	-- --	31189 (99.2)
Missing	4565 (14.0)	-- --	4565 (100.0)	

IQR, interquartile range.

Table S5. Immigrant cascade of care of individuals diagnosed with HCV in 1990-2018 and living in Quebec, Canada as of 2018, stratified by care cascade stages, with row percentages as a proportion of the previous column.

Characteristics<br><br>n (row % of previous column)	HCV diagnosed<br>n (%)	RNA tested<br>n (%)	RNA positive<br>n (%)	Genotyped<br>n (%)	Initiated treatment<br>n (%)	Assessed for SVR<br>n (%)	SVR<br>n (%)
**Total**	4862 (100.0)	4220 (86.8)	3377 (80.0)	3142 (93.0)	2388 (76.0)	1996 (83.6)	1844 (92.4)
**Birth cohort**							
<1945	487 (100.0)	371 (76.2)	316 (85.2)	267 (84.5)	165 (61.8)	132 (80.0)	121 (91.7)
1945-1965	2438 (100.0)	2130 (87.4)	1773 (83.2)	1677 (94.6)	1332 (79.4)	1116 (83.8)	1031 (92.4)
>1965	1938 (100.0)	1719 (88.7)	1288 (74.9)	1198 (93.0)	890 (74.3)	748 (84.0)	693 (92.6)
**Sex**							
Female	2177 (100.0)	1888 (86.7)	1498 (79.3)	1383 (92.3)	1018 (73.6)	857 (84.2)	791 (92.3)
Male	2685 (100.0)	2332 (86.9)	1879 (80.6)	1759 (93.6)	1370 (77.9)	1139 (83.1)	1053 (92.4)
**Region of residence**							
Urban	4367 (100.0)	3797 (86.9)	3048 (80.3)	2824 (92.7)	2150 (76.1)	1800 (83.7)	1664 (92.4)
Rural	495 (100.0)	423 (85.5)	329 (77.8)	318 (96.7)	238 (74.8)	197 (82.8)	180 (91.4)
**Social deprivation quintile**							
1 (Least deprived)	477 (100.0)	420 (88.1)	341 (81.2)	314 (92.1)	245 (78.0)	207 (84.5)	198 (95.7)
2	557 (100.0)	491 (88.2)	391 (79.6)	370 (94.6)	273 (73.8)	241 (88.3)	218 (90.5)
3	848 (100.0)	731 (86.2)	572 (78.2)	533 (93.2)	407 (76.4)	341 (83.8)	315 (92.4)
4	1282 (100.0)	1104 (86.1)	900 (81.5)	843 (93.7)	643 (76.3)	533 (82.9)	490 (91.9)
5 (Most deprived)	1699 (100.0)	1474 (86.8)	1173 (79.6)	1082 (92.2)	819 (75.7)	674 (82.3)	624 (92.6)
**Material deprivation quintile**							
1 (Least deprived)	797 (100.0)	698 (87.6)	545 (78.1)	513 (94.1)	403 (78.6)	355 (88.1)	326 (91.8)
2	826 (100.0)	718 (86.9)	590 (82.2)	556 (94.2)	412 (74.1)	354 (85.9)	330 (93.2)
3	819 (100.0)	722 (88.2)	577 (79.9)	528 (91.5)	417 (79.0)	347 (83.2)	321 (92.5)
4	889 (100.0)	772 (86.8)	632 (81.9)	593 (93.8)	451 (76.1)	374 (82.9)	340 (90.9)
5 (Most deprived)	1531 (100.0)	1311 (85.6)	1032 (78.7)	951 (92.2)	705 (74.1)	567 (80.4)	528 (93.1)
**Region of origin**							
East Asia and Pacific	915 (100.0)	765 (83.6)	602 (78.7)	562 (93.4)	439 (78.1)	353 (80.4)	341 (96.6)
South Asia	545 (100.0)	482 (88.4)	392 (81.3)	367 (93.6)	270 (73.6)	217 (80.4)	201 (92.6)
Middle East And North Africa	631 (100.0)	547 (86.7)	430 (78.6)	390 (90.7)	301 (77.2)	258 (85.7)	231 (89.5)
Sub Saharan Africa	854 (100.0)	767 (89.8)	622 (81.1)	578 (92.9)	434 (75.1)	355 (81.8)	314 (88.5)
High income Europe	783 (100.0)	683 (87.2)	547 (80.1)	520 (95.1)	408 (78.5)	363 (89.0)	342 (94.2)
Middle Income Europe And Central Asia	579 (100.0)	523 (90.3)	404 (77.2)	380 (94.1)	286 (75.3)	242 (84.6)	225 (93.0)
Latin America And Caribbean	477 (100.0)	388 (81.3)	323 (83.2)	291 (90.1)	205 (70.4)	165 (80.5)	153 (92.7)
United States, Australia and New Zealand	78 (100.0)	65 (83.3)	57 (87.7)	54 (94.7)	45 (83.3)	43 (95.6)	38 (88.4)
**Language proficiency**							
French only	1419 (100.0)	1244 (87.7)	1028 (82.6)	951 (92.5)	720 (75.7)	599 (83.2)	548 (91.5)
French and English	1066 (100.0)	948 (88.9)	724 (76.4)	684 (94.5)	532 (77.8)	452 (85.0)	420 (92.9)
English only	884 (100.0)	777 (87.9)	629 (81.0)	591 (94.0)	466 (78.8)	387 (83.0)	350 (90.4)
Neither	1493 (100.0)	1251 (83.8)	995 (79.5)	916 (92.1)	671 (73.3)	558 (83.2)	527 (94.4)
**Highest education level**							
High school or less	2445 (100.0)	2080 (85.1)	1685 (81.0)	1541 (91.5)	1149 (74.6)	943 (82.1)	884 (93.7)
Certificate/diploma	1214 (100.0)	1065 (87.7)	870 (81.7)	828 (95.2)	637 (76.9)	536 (84.1)	487 (90.9)
University/Postgraduate	1203 (100.0)	1075 (89.4)	822 (76.5)	773 (94.0)	601 (77.7)	517 (86.0)	473 (91.5)
**Years from admission to diagnosis (by quantile)**							
0-25	1276 (100.0)	1128 (88.4)	875 (77.6)	809 (92.5)	624 (77.1)	519 (83.2)	466 (89.8)
25-50	1299 (100.0)	1108 (85.3)	872 (78.7)	825 (94.6)	627 (76.0)	509 (81.2)	473 (92.9)
50-75	1154 (100.0)	994 (86.1)	805 (81.0)	753 (93.5)	580 (77.0)	484 (83.4)	445 (91.9)
75-100	1133 (100.0)	990 (87.4)	825 (83.3)	756 (91.6)	556 (73.5)	484 (87.1)	461 (95.2)

Characteristics<br><br>n (row % of previous column)	HCV diagnosed<br>n (%)	RNA tested<br>n (%)	RNA positive<br>n (%)	Genotyped<br>n (%)	Initiated treatment<br>n (%)	Assessed for SVR<br>n (%)	SVR<br>n (%)
**Decade of admission**							
1980-1989	2027 (100.0)	1738 (85.7)	1486 (85.5)	1406 (94.6)	1142 (81.2)	958 (83.9)	889 (92.8)
1990-1999	1124 (100.0)	958 (85.2)	776 (81.0)	714 (92.0)	517 (72.4)	427 (82.6)	394 (92.3)
2000-2009	1098 (100.0)	959 (87.3)	730 (76.1)	681 (93.3)	492 (72.2)	416 (84.6)	378 (90.9)
2010-2018	613 (100.0)	565 (92.2)	385 (68.1)	341 (88.6)	237 (69.5)	195 (82.3)	183 (93.8)
**People who inject drugs**							
Yes	588 (100.0)	511 (86.9)	453 (88.6)	439 (96.9)	353 (80.4)	284 (80.5)	258 (90.8)
No	4274 (100.0)	3709 (86.8)	2924 (78.8)	2703 (92.4)	2035 (75.3)	1712 (84.1)	1586 (92.6)
**Major mental health illness**							
Yes	511 (100.0)	439 (85.9)	367 (83.6)	344 (93.7)	258 (75.0)	217 (84.1)	199 (91.7)
No	4351 (100.0)	3781 (86.9)	3010 (79.6)	2798 (93.0)	2129 (76.1)	1779 (83.6)	1645 (92.5)
**Alcohol use disorder**							
Yes	116 (100.0)	101 (87.1)	90 (89.1)	81 (90.0)	61 (75.3)	50 (82.0)	45 (90.0)
No	4746 (100.0)	4119 (86.8)	3287 (79.8)	3061 (93.1)	2327 (76.0)	1946 (83.6)	1799 (92.4)
**HBV coinfection**							
Yes	159 (100.0)	113 (71.1)	79 (69.9)	72 (91.1)	56 (77.8)	45 (80.4)	40 (88.9)
No	4703 (100.0)	4107 (87.3)	3298 (80.3)	3070 (93.1)	2332 (76.0)	1951 (83.7)	1805 (92.5)
**HIV coinfection**							
Yes	137 (100.0)	132 (96.4)	115 (87.1)	114 (99.1)	102 (89.5)	93 (91.2)	84 (90.3)
No	4725 (100.0)	4088 (86.5)	3263 (79.8)	3029 (92.8)	2286 (75.5)	1903 (83.2)	1760 (92.5)
**Decompensated cirrhosis**							
Yes	144 (100.0)	133 (92.4)	118 (88.7)	114 (96.6)	97 (85.1)	82 (84.5)	73 (89.0)
No	4718 (100.0)	4087 (86.6)	3259 (79.7)	3028 (92.9)	2291 (75.7)	1914 (83.5)	1771 (92.5)
**Hepatocellular carcinoma**							
Yes	77 (100.0)	73 (94.8)	73 (100.0)	72 (98.6)	67 (93.1)	52 (77.6)	45 (86.5)
No	4785 (100.0)	4147 (86.7)	3304 (79.7)	3070 (92.9)	2321 (75.6)	1944 (83.8)	1799 (92.5)
**Diabetes**							
Yes	454 (100.0)	395 (87.0)	354 (89.6)	322 (91.0)	252 (78.3)	210 (83.3)	194 (92.4)
No	4408 (100.0)	3825 (86.8)	3023 (79.0)	2820 (93.3)	2136 (75.7)	1787 (83.7)	1651 (92.4)
**Elixhauser comorbidity index (modified)**							
Yes	234 (100.0)	202 (86.3)	166 (82.2)	158 (95.2)	131 (82.9)	100 (76.3)	93 (93.0)
No	4628 (100.0)	4019 (86.8)	3211 (79.9)	2984 (92.9)	2257 (75.6)	1896 (84.0)	1751 (92.4)
**Period of HCV diagnosis**							
1990-2013	3859 (100.0)	3310 (85.8)	2722 (82.2)	2573 (94.5)	1990 (77.3)	1670 (83.9)	1530 (91.6)
2014-2018	1003 (100.0)	910 (90.7)	655 (72.0)	569 (86.9)	398 (69.9)	326 (81.9)	314 (96.3)

Table S6. Predictors of not having received RNA testing or initiated treatment as of 2018 among immigrants, and changes since 2013, using generalized estimating equation logistic regression analysis.

Characteristics[a]	Model A: Left behind of RNA testing				Model B: Left behind of treatment initiation			
	Overall effect: 2018		Interaction terms[b]		Overall effect: 2018		Interaction terms[b]	
	aOR (95% CI)	P value	2013 aOR (95% CI)	2018 aOR (95% CI)	aOR (95% CI)	P value	2013 aOR (95% CI)	2018 aOR (95% CI)
**Sex**								
Male (Ref)	1 (Ref)		--	--	--	--	--	--
Female	0.97 (0.81-1.15)	0.711	--	--	--	--	1.29 (1.11-1.51)[¥]	1.06 (0.91-1.23)
**Birth cohort**								
<1945	1.79 (1.33-2.40)	0.001	--	--	5.11 (4.03-6.49)	<.0001	--	--
1945-1965	0.96 (0.80-1.17)	0.710	--	--	1.11 (0.95-1.29)	0.206	--	--
>1965 (Ref)	1 (Ref)		--	--	1 (Ref)	1 (Ref)	--	--
**PWID**								
No (Ref)	1 (Ref)		--	--	--	--	1 (Ref)	1 (Ref)
Yes	0.92 (0.64-1.32)	0.651	--	--	--	--	1.35 (1.04-1.76)[*]	0.80 (0.60-1.05)
**Region of origin**								
East Asia And Pacific	1.19 (0.87-1.62)	0.266	--	--	--	--	1.15 (0.88-1.50)	0.87 (0.67-1.14)
South Asia	0.87 (0.59-1.27)	0.457	--	--	--	--	0.87 (0.62-1.23)	1.13 (0.82-1.55)
Middle East & North Africa	0.91 (0.65-1.28)	0.593	--	--	--	--	1.03 (0.77-1.37)	1.09 (0.83-1.44)
Sub Saharan Africa	0.92 (0.65-1.30)	0.644	--	--	--	--	1.30 (0.95-1.78)	1.04 (0.77-1.42)
High Income Regions[c] (Ref)	1 (Ref)		--	--	--	--	1 (Ref)	1 (Ref)
Middle Income Europe & Central Asia	0.84 (0.60-1.18)	0.312	--	--	--	--	1.25 (0.91-1.72)	1.04 (0.77-1.40)
Latin America and Caribbean	1.50 (1.02-2.22)	0.040	--	--	--	--	1.45 (1.07-1.98)[*]	1.46 (1.07-2.00)[*]
**Region of residence**								
Rural	1.31 (1.01-1.69)	0.045	--	--	0.88 (0.70-1.11)	0.279	--	--
Urban (Ref)	1 (Ref)		--	--	1 (Ref)		--	--
**Material deprivation index**								
1 (Most privileged) (Ref)	1 (Ref)		--	--	--	--	1 (Ref)	1 (Ref)
2	1.20 (0.85-1.68)	0.299	--	--	--	--	1.26 (0.96-1.66)	1.15 (0.89-1.49)
3	1.11 (0.78-1.57)	0.567	--	--	--	--	1.17 (0.90-1.53)	1.04 (0.80-1.35)
4	1.21 (0.89-1.64)	0.224	--	--	--	--	1.08 (0.81-1.44)	1.07 (0.82-1.41)
5 (Most deprived)	1.33 (0.98-1.80)	0.071	--	--	--	--	1.66 (1.30-2.12)	1.24 (0.98-1.57)
**Social deprivation index**								
1 (Most privileged)	1 (Ref)		--	--	--	--	--	--
2	0.97 (0.64-1.48)	0.904	--	--	0.88 (0.70-1.11)	0.279	--	--
3	1.17 (0.79-1.72)	0.433	--	--	1.16 (0.89-1.50)	0.272	--	--
4	1.14 (0.81-1.58)	0.454	--	--	1.26 (0.97-1.63)	0.088	--	--
5 (Most deprived) (Ref)	1.13 (0.80-1.60)	0.487	--	--	1.37 (1.07-1.76)	0.013	--	--
**Elixhauser comorbidity index (modified)**								
No (Ref)	1 (Ref)		--	--	1 (Ref)		--	--
Yes	0.92 (0.58-1.45)	0.713	--	--	1.16 (0.84-1.62)	0.359	--	--
**Diabetes**								
No (Ref)	1 (Ref)		--	--	--	--	1 (Ref)	1 (Ref)
Yes	1.03 (0.76-1.40)	0.850	--	--	--	--	0.78 (0.60-1.01)	1.11 (0.90-1.38)
**Major mental health diagnosis**								
No (Ref)	1 (Ref)		--	--	1 (Ref)		--	--
Yes	0.99 (0.74-1.34)	0.953	--	--	1.24 (0.99-1.55)	0.063	--	--

Characteristics[a]	Model A: Left behind of RNA testing				Model B: Left behind of treatment initiation			
	Overall effect: 2018		Interaction terms[b]		Overall effect: 2018		Interaction terms[b]	
	aOR (95% CI)	P value	2013 aOR (95% CI)	2018 aOR (95% CI)	aOR (95% CI)	P value	2013 aOR (95% CI)	2018 aOR (95% CI)
**Alcohol use disorder**								
No (Ref)	1 (Ref)		--	--	1 (Ref)		--	--
Yes	1.16 (0.57-2.35)	0.686	--	--	1.50 (0.94-2.37)	0.086	--	--
**HBV coinfection**								
No (Ref)	1 (Ref)		--	--	1 (Ref)		--	--
Yes	2.59 (1.70-3.93)	<0.001	--	--	1.31 (0.85-2.03)	0.227	--	--
**HIV coinfection**								
No (Ref)	--	--	--	--	--	--	1 (Ref)	1 (Ref)
Yes	0.27 (0.12-0.62)	0.002	--	--	--	--	1.29 (0.79-2.12)	0.41 (0.24-0.71)*
**Decompensated cirrhosis**								
No (Ref)	--	--	--	--	1 (Ref)		--	--
Yes	0.61 (0.29-1.27)	0.185	--	--	0.94 (0.59-1.50)	0.809	--	--
**Hepatocellular carcinoma**								
No (Ref)	--	--	--	--	--	--	1 (Ref)	1 (Ref)
Yes	0.34 (0.08-1.34)	0.123	--	--	--	--	1.26 (0.59-2.68)	0.41 (0.19-0.89)*

Effects for 2013 shown only for characteristics with a significant (p<0.05) change from 2013 to 2018.

aOR, adjusted odds ratios. -- Left blank on purpose. *p<0.05. ¥p<0.01. ǂp<0.001.

[a]Characteristics as of 2018; model also adjusted for calendar year of HCV diagnosis and immigration category.

[b]Adjusted odds ratios generated from interaction terms of each variable with the care cascade calendar year (i.e. 2013 or 2018). Only interaction terms with p<0.05 were included in the final model and are shown in the table above.

Table S7. Sensitivity analysis: predictors of time to RNA testing and treatment initiation among individuals diagnosed with HCV in Quebec, Canada, 1990-2018 using Fine-Gray subdistribution competing risk hazard regression analysis.

Characteristics[a]	HCV diagnosis to RNA testing		RNA positive to treatment initiation	
	aHR (95% CI)	P value	aHR (95% CI)	P value
**Sex**				
Male (Ref)	1 (Ref)		1 (Ref)	
Female	1.00 (0.98-1.03)	0.773	0.96 (0.93-1.00)	0.048
**Birth cohort**				
<1945	0.68 (0.64-0.72)	<0.001	0.29 (0.27-0.32)	<0.001
1945-1965	0.95 (0.93-0.97)	<0.001	0.79 (0.77-0.82)	<0.001
>1965 (Ref)	1 (Ref)		1 (Ref)	
**People who inject drugs[b]**				
No (Ref)	1 (Ref)		1 (Ref)	
Yes	0.81 (0.76-0.87)	<0.001	0.66 (0.62-0.71)	<0.001
**People who inject drugs*time[b]**	1.08 (1.05-1.11)	<0.001	1.04 (1.03-1.05)	<0.001
**Immigrant status**				
Non-immigrant (Ref)	1 (Ref)		1 (Ref)	
Immigrant	1.12 (1.09-1.15)	<0.001	1.42 (1.35-1.48)	<0.001
**Region of residence**				
Urban	1.14 (1.11-1.17)	<0.001	0.99 (0.95-1.03)	0.646
Rural (Ref)	1 (Ref)		1 (Ref)	
**Material deprivation index**				
1 (Most privileged) (Ref)	1 (Ref)		1 (Ref)	
2	0.94 (0.90-0.98)	0.002	0.98 (0.92-1.04)	0.442
3	0.95 (0.91-0.99)	0.014	0.94 (0.88-0.99)	0.029
4	0.94 (0.91-0.98)	0.002	0.94 (0.89-1.00)	0.037
5 (Most deprived)	0.90 (0.87-0.93)	<0.001	0.87 (0.82-0.92)	<0.001
**Social deprivation index**				
1 (Most privileged) (Ref)	1 (Ref)		1 (Ref)	

Characteristics[a]	HCV diagnosis to RNA testing		RNA positive to treatment initiation	
	aHR (95% CI)	P value	aHR (95% CI)	P value
2	1.01 (0.97-1.06)	0.641	1.00 (0.93-1.07)	0.933
3	0.98 (0.94-1.03)	0.404	0.96 (0.90-1.03)	0.282
4	0.95 (0.91-1.00)	0.038	0.91 (0.85-0.96)	0.002
5 (Most deprived)	0.92 (0.89-0.96)	<0.001	0.86 (0.81-0.91)	<0.001
**Elixhauser comorbidity index (modified)[c]**				
No (Ref)	1 (Ref)		1 (Ref)	
Yes	0.94 (0.89-0.99)	0.031	0.95 (0.89-1.02)	0.166
**Diabetes[d]**				
No (Ref)	1 (Ref)		1 (Ref)	
Yes	0.97 (0.92-1.02)	0.188	1.33 (1.27-1.39)	<0.001
**Major mental health diagnosis[d]**				
No (Ref)	1 (Ref)		1 (Ref)	
Yes	1.11 (1.08-1.14)	<0.001	1.14 (1.09-1.19)	<0.001
**Alcohol use disorder[d]**				
No (Ref)	1 (Ref)		1 (Ref)	
Yes	0.94 (0.90-0.97)	<0.001	0.68 (0.64-0.71)	<0.001
**HBV coinfection[d]**				
No (Ref)	1 (Ref)		1 (Ref)	
Yes	0.82 (0.76-0.89)	<0.001	0.96 (0.88-1.04)	0.316
**HIV coinfection[d]**				
No (Ref)	1 (Ref)		1 (Ref)	
Yes	1.28 (1.21-1.35)	<0.001	1.18 (1.11-1.25)	<0.001
**Decompensated cirrhosis[d]**				
No (Ref)	1 (Ref)		1 (Ref)	
Yes	1.38 (1.30-1.46)	<0.001	1.70 (1.62-1.78)	<0.001
**Hepatocellular carcinoma[d]**				
No (Ref)	1 (Ref)		1 (Ref)	
Yes	0.63 (0.52-0.75)	<0.001	0.62 (0.54-0.71)	<0.001

aHR, adjusted hazard ratios. Model also adjusted for calendar year of HCV diagnosis.

[a]Characteristics at the time of HCV diagnosis, unless otherwise specified. [b]Non-proportional hazards addressed by adding an interaction with time. [c]Diagnostic codes for diabetes, mental health illness, alcohol and drug consumption disorders, HIV, and liver-disease excluded. [d]Time-varying covariates.

Table S8. Sensitivity analysis: predictors of time to RNA testing and treatment initiation among individuals diagnosed with HCV in Quebec, Canada, 1990-2018 using Fine-Gray subdistribution competing risk hazard regression analysis (immigrants stratified by World Bank region of origin).

Characteristics[1]	HCV diagnosis to RNA testing		RNA positive to treatment initiation	
	aHR (95% CI)	P value	aHR (95% CI)	P value
**Sex**				
Male (Ref)	1 (Ref)		1 (Ref)	
Female	1.00 (0.98-1.03)	0.770	0.96 (0.93-1.00)	0.054
**Birth cohort**				
<1945	0.68 (0.64-0.72)	<0.001	0.30 (0.27-0.32)	<0.001
1945-1965	0.95 (0.93-0.98)	<0.001	0.80 (0.77-0.82)	<0.001
>1965 (Ref)	1 (Ref)		1 (Ref)	
**People who inject drugs[2]**				
No (Ref)	1 (Ref)		1 (Ref)	
Yes	0.81 (0.76-0.87)	<0.001	0.66 (0.62-0.71)	<0.001
**People who inject drugs*time[2]**				
(Continuous)	1.08 (1.05-1.11)	<0.001	1.04 (1.03-1.05)	<0.001
**Region of birth**				
Canada (Ref)	1 (Ref)		1 (Ref)	
East Asia and Pacific	1.09 (1.02-1.17)	0.013	1.43 (1.30-1.57)	<0.001
High Income Regions	1.10 (1.05-1.16)	<0.001	1.41 (1.29-1.54)	<0.001
Latin America and Caribbean	0.95 (0.86-1.05)	0.308	1.16 (1.01-1.33)	0.041
Middle East & North Africa	1.17 (1.09-1.25)	<0.001	1.52 (1.35-1.72)	<0.001
South Asia	1.16 (1.08-1.24)	<0.001	1.43 (1.26-1.63)	<0.001
Sub Saharan Africa	1.20 (1.12-1.29)	<0.001	1.42 (1.28-1.59)	<0.001
Middle Income Europe & Central Asia	1.19 (1.13-1.26)	<0.001	1.52 (1.35-1.70)	<0.001
**Region of residence**				
Urban	1.14 (1.11-1.17)	<0.001	0.99 (0.95-1.03)	0.625

Characteristics[1]	HCV diagnosis to RNA testing		RNA positive to treatment initiation	
	aHR (95% CI)	P value	aHR (95% CI)	P value
Rural (Ref)	1 (Ref)		1 (Ref)	
**Material deprivation index**				
1 (Most privileged) (Ref)	1 (Ref)		1 (Ref)	
2	0.94 (0.90-0.98)	0.002	0.98 (0.92-1.04)	0.457
3	0.95 (0.91-0.99)	0.012	0.94 (0.88-0.99)	0.028
4	0.94 (0.91-0.98)	0.002	0.94 (0.89-1.00)	0.037
5 (Most deprived)	0.90 (0.87-0.93)	<0.001	0.87 (0.82-0.92)	<0.001
**Social deprivation index**				
1 (Most privileged) (Ref)	1 (Ref)		1 (Ref)	
2	1.01 (0.97-1.06)	0.659	1.00 (0.93-1.07)	0.944
3	0.98 (0.94-1.03)	0.393	0.96 (0.90-1.03)	0.288
4	0.95 (0.91-1.00)	0.038	0.91 (0.85-0.97)	0.002
5 (Most deprived)	0.92 (0.88-0.96)	<0.001	0.86 (0.81-0.91)	<0.001
**Year of HCV diagnosis**				
(Continuous)	1.06 (1.06-1.06)	<0.001	1.04 (1.03-1.04)	<0.001
**Elixhauser comorbidity index (modified)[3]**				
No (Ref)	1 (Ref)		1 (Ref)	
Yes	0.94 (0.90-0.99)	0.032	0.95 (0.89-1.02)	0.173
**Diabetes[4]**				
No (Ref)	1 (Ref)		1 (Ref)	
Yes	0.97 (0.92-1.02)	0.181	1.33 (1.27-1.39)	<0.001
**Major mental health diagnosis[4]**				
No (Ref)	1 (Ref)		1 (Ref)	
Yes	1.11 (1.08-1.14)	<0.001	1.14 (1.09-1.19)	<0.001
**Alcohol use disorder [4]**				
No (Ref)	1 (Ref)		1 (Ref)	
Yes	0.94 (0.90-0.97)	<0.001	0.68 (0.64-0.71)	<0.001
**HBV coinfection[4]**				
No (Ref)	1 (Ref)		1 (Ref)	
Yes	0.82 (0.76-0.88)	<0.001	0.96 (0.88-1.04)	0.307
**HIV coinfection[4]**				
No (Ref)	1 (Ref)		1 (Ref)	
Yes	1.28 (1.21-1.35)	<0.001	1.19 (1.12-1.26)	<0.001
**Decompensated cirrhosis[4]**				
No (Ref)	1 (Ref)		1 (Ref)	
Yes	1.38 (1.30-1.47)	<0.001	1.70 (1.62-1.78)	<0.001
**Hepatocellular carcinoma[4]**				
No (Ref)	1 (Ref)		1 (Ref)	
Yes	0.62 (0.52-0.75)	<0.001	0.62 (0.54-0.71)	<0.001

aHR, adjusted hazard ratios.

[1]Characteristics at the time of HCV diagnosis, unless otherwise specified. [2]Non-proportional hazards addressed by adding an interaction with time. [3]Diagnostic codes for diabetes, mental health illness, alcohol and drug consumption disorders, HIV, and liver-disease excluded. [4]Time-varying covariates.

Table S9. Sensitivity analysis: predictors of time to RNA testing and treatment initiation among immigrants diagnosed with HCV in Quebec, Canada, 1990-2018 using Fine-Gray subdistribution competing risk hazard regression analysis.

Characteristics[a]	HCV diagnosis to RNA testing		RNA positive to treatment initiation	
	aHR (95% CI)	P value	aHR (95% CI)	P value
**Sex**				
Male (Ref)	1 (Ref)		1 (Ref)	
Female	1.00 (0.94-1.06)	0.998	0.91 (0.84-0.98)	0.018
**Birth cohort**				
<1945	0.70 (0.62-0.79)	<0.001	0.38 (0.32-0.46)	<0.001
1945-1965	1.00 (0.95-1.06)	0.925	0.97 (0.89-1.05)	0.437
>1965 (Ref)	1 (Ref)		1 (Ref)	
**People who inject drugs[b]**				
No (Ref)	1 (Ref)		1 (Ref)	
Yes	0.83 (0.71-0.97)	0.018	0.66 (0.53-0.83)	<0.001
**People who inject drugs*time**	1.05 (0.97-1.13)	0.246	1.06 (1.03-1.09)	<0.001
**Region of residence**				
Urban	1.15 (1.03-1.29)	0.011	1.03 (0.90-1.19)	0.647
Rural (Ref)	1 (Ref)		1 (Ref)	
**Material deprivation index**				
1 (Most privileged)	1 (Ref)		1 (Ref)	
2	0.97 (0.89-1.05)	0.435	0.96 (0.83-1.09)	0.507
3	0.97 (0.87-1.07)	0.544	0.94 (0.83-1.08)	0.394

Characteristics[a]	HCV diagnosis to RNA testing		RNA positive to treatment initiation	
	aHR (95% CI)	P value	aHR (95% CI)	P value
4	0.96 (0.87-1.04)	0.313	0.98 (0.86-1.11)	0.728
5 (Most deprived) (Ref)	0.95 (0.88-1.04)	0.258	0.91 (0.80-1.03)	0.122
**Social deprivation index**				
1 (Most privileged)	1 (Ref)		1 (Ref)	
2	1.04 (0.92-1.17)	0.566	0.98 (0.83-1.16)	0.824
3	0.99 (0.87-1.12)	0.819	1.02 (0.87-1.19)	0.830
4	0.95 (0.84-1.08)	0.455	1.00 (0.86-1.15)	0.948
5 (Most deprived) (Ref)	0.97 (0.86-1.09)	0.640	0.94 (0.82-1.08)	0.399
**Region of birth**				
High Income Regions (Ref)	1 (Ref)		1 (Ref)	
East Asia and Pacific	0.96 (0.87-1.07)	0.455	1.05 (0.92-1.21)	0.449
Latin America and Caribbean	0.86 (0.74-1.00)	0.046	0.82 (0.69-0.98)	0.027
Middle East & North Africa	1.05 (0.95-1.16)	0.385	1.06 (0.91-1.23)	0.488
South Asia	1.07 (0.95-1.20)	0.298	1.06 (0.88-1.26)	0.546
Sub Saharan Africa	1.06 (0.97-1.17)	0.204	0.98 (0.84-1.14)	0.754
Middle Income Europe & Central Asia	1.06 (0.96-1.16)	0.252	1.06 (0.91-1.23)	0.482
**Immigration category**				
Economic (Ref)	1 (Ref)		1 (Ref)	
Family	0.94 (0.87-1.01)	0.102	0.95 (0.86-1.04)	0.266
Refugee	0.99 (0.91-1.07)	0.766	0.92 (0.83-1.03)	0.154
**Years from admission to diagnosis (continuous)**	1.00 (0.99-1.00)	0.719	1.00 (0.99-1.00)	0.159
**Year of HCV diagnosis (continuous)**	1.05 (1.04-1.06)	<0.001	1.06 (1.05-1.07)	<0.001
**Diabetes**				
No (Ref)	1 (Ref)		1 (Ref)	
Yes	1.06 (0.95-1.18)	0.317	1.26 (1.14-1.39)	<0.001
**Elixhauser comorbidity index (modified)**				
No (Ref)	1 (Ref)		1 (Ref)	
Yes	0.93 (0.74-1.18)	0.567	0.98 (0.80-1.20)	0.852
**Major mental health diagnosis[b]**				
No (Ref)	1 (Ref)		1 (Ref)	
Yes	1.12 (1.01-1.25)	0.027	1.06 (0.96-1.16)	0.283
**Alcohol use disorder [b]**				
No (Ref)	1 (Ref)		1 (Ref)	
Yes	0.98 (0.76-1.26)	0.849	0.64 (0.51-0.81)	<0.001
**HBV coinfection[b]**				
No (Ref)	1 (Ref)		1 (Ref)	
Yes	0.76 (0.60-0.96)	0.021	0.99 (0.80-1.21)	0.891
**HIV coinfection[b]**				
No (Ref)	1 (Ref)		1 (Ref)	
Yes	1.22 (1.04-1.43)	0.013	1.33 (1.11-1.60)	0.002
**Decompensated cirrhosis[b]**				
No (Ref)	1 (Ref)		1 (Ref)	
Yes	1.36 (1.13-1.64)	0.001	1.58 (1.42-1.75)	<0.001
**Hepatocellular carcinoma[b]**				
No (Ref)	1 (Ref)		1 (Ref)	
Yes	0.71 (0.45-1.14)	0.155	0.62 (0.46-0.83)	0.001

aHR, adjusted hazard ratios. [a]Characteristics at the time of HCV diagnosis, unless otherwise specified. [b]Time-varying covariates.

Figure S1. Flowchart of the identification of hepatitis C diagnosis and cohort creation.

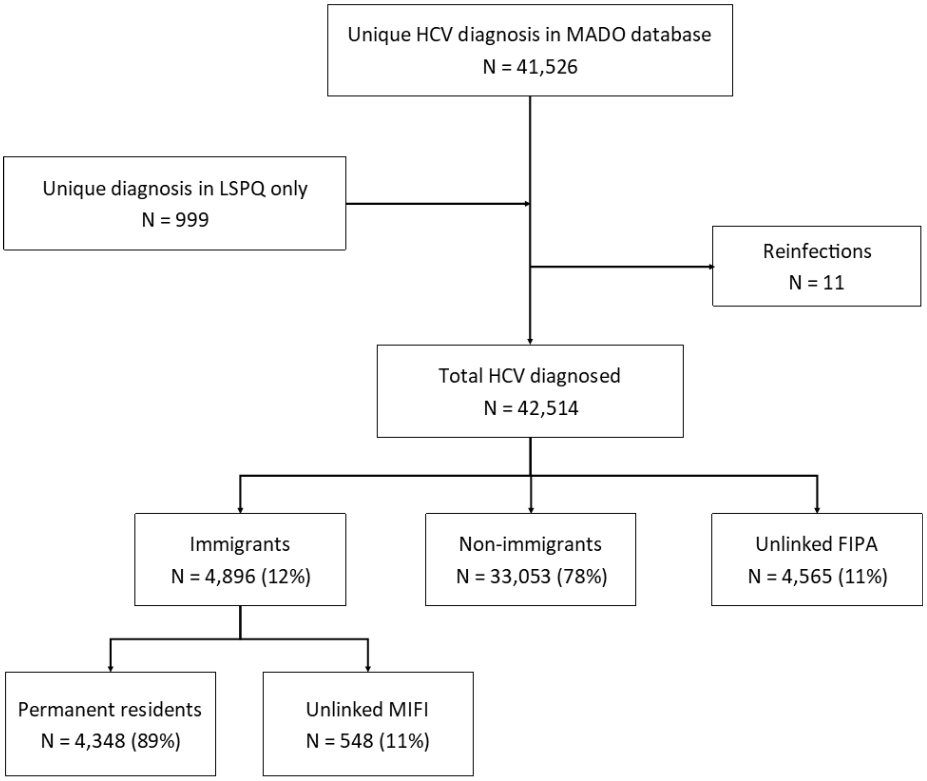

Abbreviations: MADO, public health reportable diseases database ("Maladies à déclaration obligatoire"); LSPQ, provincial reference laboratory database ("Laboratoire de santé publique du Québec"); FIPA, database for province healthcare covered individuals ("Fichier d'inscription des personnes assurées"); MIFI, landed immigrants database ("Ministry of Immigration, Frenchisation and Integration").

Care cascade backfilling:

The HCV care cascade cohort included all confirmed HCV antibody diagnoses reported to the public health reportable diseases database (MADO) and all the confirmed HCV RNA diagnoses present in the provincial reference laboratory database (LSPQ). As seen in **Figure S1** above, 999 (2.3%) of all HCV diagnoses in the cohort were only present in LSPQ but not MADO; the "HCV diagnosed" step of the care cascade was backfilled for these individuals. We also backfilled care cascade steps for individuals with an observed genotype test or antiviral treatment dispensation, which accounted for 1.7% of all HCV diagnoses in the cohort.

Figure S2. Intersectionality of priority groups among individuals diagnosed with HCV since 1990 and alive in 2018, Quebec, Canada.

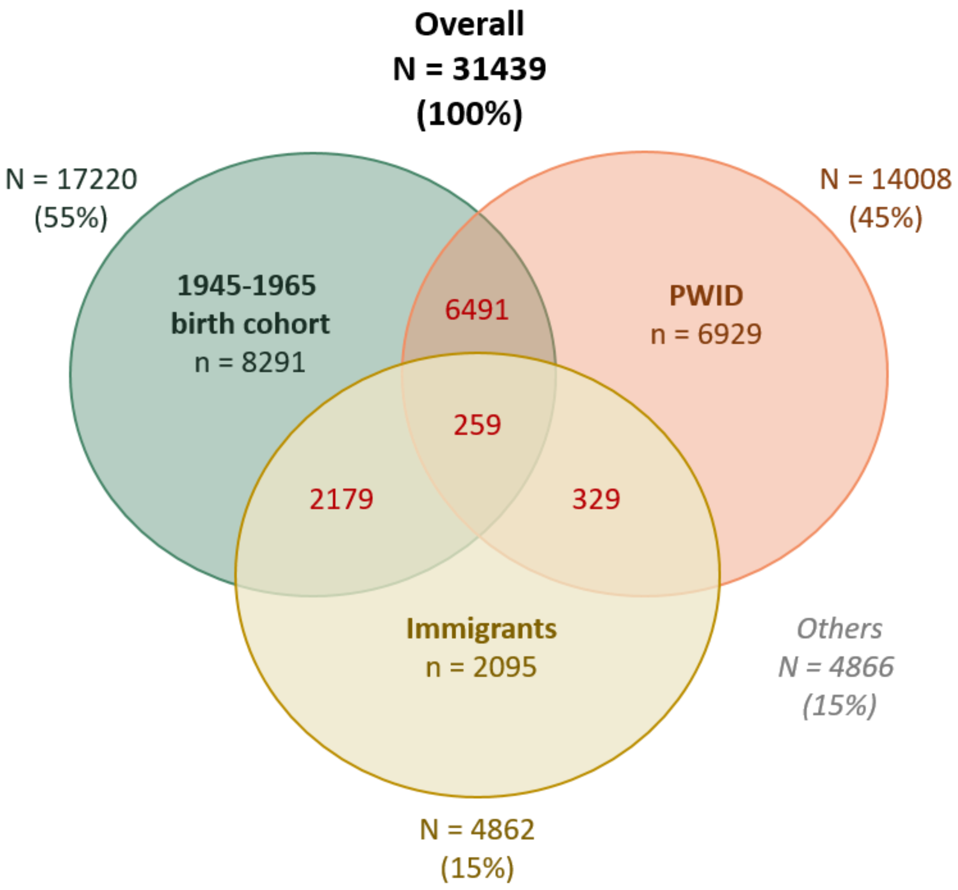

Figure S3. Time-varying hazard for people who inject drugs (PWID) diagnosed with HCV in Quebec, Canada, 1990-2018. Shown are adjusted hazard ratios (aHR) with 95% confidence intervals (CI) over time from (A) HCV diagnosis to RNA testing and from (B) RNA positive to treatment initiation. These data result from the inclusion of an interaction of the PWID variable and time to event in the Fine-Gray subdistribution competing hazard regression model (shown in Table 2).

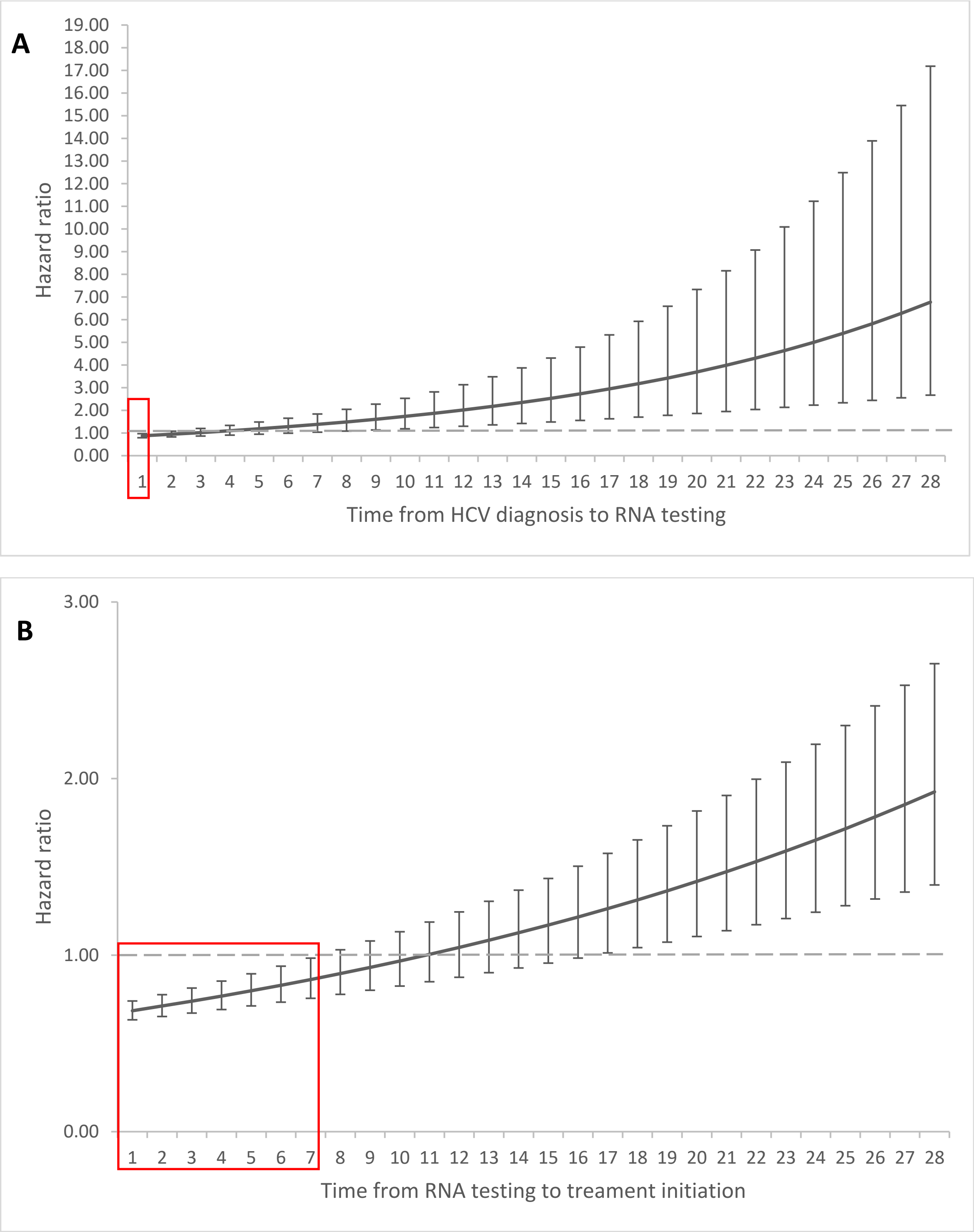

Supplementary References

1. Janjua NZ, Islam N, Kuo M, Yu A, Wong S, Butt ZA, et al. Identifying injection drug use and estimating population size of people who inject drugs using healthcare administrative datasets. Int J Drug Policy. 2018;55:31-9.
2. Greenwald Z, Feld J, Werb D, et al. Validation of case-ascertainment algorithms to identify people who inject drugs in health administrative data in Ontario. Canadian Liver Journal [Canadian Liver Meeting]. 2022;CLM22-ABS- 1037.
3. Bruneau J, Lamothe F, Soto J, Lachance N, Vincelette J, Vassal A, et al. Sex-specific determinants of HIV infection among injection drug users in Montreal. Cmaj. 2001;164(6):767-73.
4. Bruneau J, Roy E, Arruda N, Zang G, Jutras-Aswad D. The rising prevalence of prescription opioid injection and its association with hepatitis C incidence among street-drug users. Addiction. 2012;107(7):1318-27.
5. Jacka B, Larney S, Degenhardt L, Janjua N, Hoj S, Krajden M, et al. Prevalence of Injecting Drug Use and Coverage of Interventions to Prevent HIV and Hepatitis C Virus Infection Among People Who Inject Drugs in Canada. Am J Public Health. 2020;110(1):45-50.
6. Steyerberg EW, Vickers AJ, Cook NR, Gerds T, Gonen M, Obuchowski N, et al. Assessing the performance of prediction models: a framework for traditional and novel measures. Epidemiology. 2010;21(1):128-38.
7. Action Hepatitis Canada. Progress Towards Viral Hepatitis Elimination By 2030 In Canada. Internet. Toronto, ON: AHC; 2021.
8. Canada I. IQVIA Canada 2022 [Available from: https://www.iqvia.com/?utm_source=google&utm_medium=cpc&utm_campaign=2021_iqvia_brand&utm_content=93726100272&utm_term=%2Biqvia&gclid=CjwKCAjw3qGYBhBSEiwAcnTRLqOxk4VGbKZlsosfPcXdSDuQVilAa7E0FLgLTJBewELxWoe8hq4mgRoCITQQAvD_BwE.]
